# Supplementary material for: Recurrent Dissemination of SARS-CoV-2 Through the Uruguayan–Brazilian Border
Source: Front Microbiol. 2021 May 28;12:653986. doi: 10.3389/fmicb.2021.653986 (PMC8195593; doi:10.3389/fmicb.2021.653986)
Supplement: Supplementary file 1 [file Data_Sheet_1.pdf]

SUPPLEMENTARY MATERIALS

Section 1. Materials and Methods

1.1. SARS-CoV-2 RNA identification from samples

In the UdelaR Labs Network for SARS-CoV-2 molecular detection (CENUR Regional Norte-Salto, CURE Regional Este-Rocha, Campus Tacuarembó, Uruguay) previously described, located in Salto, Rocha and Tacuarembó cities, the viral RNA extraction was performed directly from samples (virus transport media containing the swab collected) by using the Viral RNA Isolation Kit (Liferiver Bio-Tech Corp., San Diego, USA), according to the manufacturer's instructions. The viral RNA extracted was used as a template in a OneStep RT-qPCR kit *COVID-19 RT-PCR Real TM Fast* (UdelaR, IPMON and ATGen S.R.L., Montevideo, Uruguay) to test the presence of SARS-CoV-2 RNA in the samples, according to the manufacturer's instructions. Sanatorio Americano Montevideo (SASA, Laboratory of Molecular Biology) used on the one hand, Coronavirus COVID-19 genesig® Real-Time PCR assay and on the other, GeneFinder Covid-19 Plus RealAmp Kit, according to manufacturer's instructions. In Brazil in both institutions, the viral RNA was obtained by QIAamp Viral RNA Mini kit (QIAGEN, Hilden, Germany) or automatedly Perkin-Elmer Chemagic machine/chemistry using 140 µl or 300 µl of the sample, respectively, according to the manufacturer's instructions. SARS-CoV-2 positive cases were confirmed by real-time RT-PCR assays using the SARS-CoV-2 Molecular E/RP Kit (Biomanguinhos, Rio de Janeiro, Brazil) based on the primers previously designed by[1]

1.2. Reverse transcription and SARS-CoV-2 whole-genome amplification

Total RNA from positive samples was reverse transcribed using SuperScript™ IV First Strand Synthesis System (Invitrogen, Carlsbad, CA, USA), according to the manufacturer's instructions. Two multiplex PCR reactions with the primer scheme (Pool A = nine amplicons and Pool B = eight amplicons) were performed for building long (~2kb) amplicon libraries to recover SARS-CoV-2 genomes as previously described by Resende and co-workers[2], by using the Q5® High-Fidelity DNA Polymerase (New England Biolabs, Ipswich, MA, USA), according to the manufacturer's instructions. When SARS-CoV-2 genome amplification was unsuccessful with the long 2kb amplicons strategy, a second strategy was used. In this case SARS-CoV-2 genomes were recovered by building short (~400pb) amplicon libraries as previously described by Quick and co-workers[3], using the hCoV-19 primer scheme V3 (which can be obtained under https://github.com/artic-network/primer-schemes/tree/master/nCoV-2019/V3). Both protocols (long and

short amplicons libraries) are based on the amplicon tiling strategy described previously by Quick and co-workers[4]. Both pools were mixed and the amplicons were purified using Agencourt AMPure XP beads (Beckman Coulter™, Brea, CA, USA) and a quality control was performed to measure the quantity of DNA using the Qubit™ dsDNA BR Assay Kit (Invitrogen) and Qubit Fluorometric Quantification.

1.3. SARS-CoV-2 whole-genome sequencing

Different sequencing methods were used to produce the genomes for this study, such as Illumina, Nanopore and Ion Torrent. ONT libraries were prepared by using the Ligation Sequencing Kit (SQK-LSK109) and Native Barcoding Expansion (EXP-NBD104 and EXP-NBD114), both from ONT (Oxford Nanopore Technologies, United Kingdom). The NEBNext Ultra II End Repair/dA-Tailing Module and NEBNext Ultra II Ligation Module were used to ligate barcodes and sequence adapters to each sample (New England Biolabs, Ipswich, MA, USA), according to the manufacturer's instructions. Negative controls using H2O as template (no RNA) in the RT step were added as amplification controls and were kept in the rest of the process as sequencing controls. We used MinION sequencing platforms (Oxford Nanopore Technologies Ltd., Oxford, UK) as described in detail elsewhere[5]. Illumina short reads DNA libraries were generated from the pooled amplicons using Nextera XT DNA Sample Preparation Kit (Illumina, San Diego, CA, USA) according to the manufacturer specifications. Library sizes were evaluated using a 4200 TapeStation System (Agilent, Santa Clara, USA). Samples were then sequenced (pair-end) (Micro V2, 300 cycles) on a MiSeq equipment (Illumina, San Diego, USA) in around 18 hours. Ion Torrent: 100 ng of each purified PCR products were used to produce Ion Torrent™ compatible sequencing libraries, using NEBNext® Fast DNA Library Prep Set for Ion Torrent™ (#E6270L, New England Biolabs, Inc) and Ion Xpress™ Barcode Adapters (#4474517, Ion Torrent™) following manufacturer instructions. Following Qubit™ Fluorometer quantification with Qubit™ dsDNA HS Assay Kit, libraries were diluted and combined in a equimolar fashion to obtain 25 µl of 50 pM the input for the sequencing template preparation. Template preparation and Ion 530™ Chip loading were conducted with the Ion Chef™ Instrument, with the Ion 510™ & Ion 520™ & Ion 530™ Kit – Chef (#A34019, Ion Torrent™), following manufacturer instructions. Sequencing was performed in the Ion GeneStudio™ S5 System™, using Ion 510™ & Ion 520™ & Ion 530™ Kit – Chef (#A34019, Ion Torrent™), following manufacturer instructions.

1.4. SARS-CoV-2 whole-genome consensus sequences

Each of the sequencing platforms used generated fastq files analyzed by different methods to obtain final consensus sequence.

The raw data from the MinION was analyzed with the pipeline developed by the COVID-19 Genomics UK consortium[6], which is based on the Artic Network bioinformatic pipeline[7]. The pipeline was embedded within Nextflow as in[8] with minor modifications to fit local infrastructure[9].

Within that pipeline different combinations of parameters were used. For the demultiplexing step with Guppy v3.6.0 (https://community.nanoporetech.com) we asked for both barcodes to be present (at both ends with min_score 50 and min_score_rear_override 40). Alternatively, we requested one barcode with a higher score (min_score 60). Careful inspection of results indicated that relaxing Guppy parameters decreased N content in the final sequence without introducing any variants.

Ion Torrent reads were mapped to the reference genome using Bowtie 2[10] allowing local alignments. Finally, bcftools was used for SNP calling (mpileup), SNP filtering (minimum quality of 20 and filtering adjacent indels within 5 bases) and to build the consensus sequences (consensus function). Positions of interest (about 20 bases around synapomorphic sites) were manually inspected to resolve undetermined bases.

In Brazilian Institutions, demultiplexed fastq files generated by Illumina or IonTorrent sequencing were used as the input for the analysis. Reads were trimmed based on quality scores with a cutoff of Q30, in order to remove low quality regions and adapter sequences were filtered. Following standard pre-processing steps, reads were mapped to the hCoV-19/Wuhan/Hu-1/2019 strain (GISAID accession number EPI_ISL_402125). Duplicate reads were removed from the alignment and the consensus sequence called at a threshold of 10x. The entire workflow was carried out in CLC Genomics Workbench software version 20.0 (https://digitalinsights.qiagen.com).

1.5 Maximum likelihood phylogenetic analyses

All full-length B.1.1.28 (n = 275) and B.1.1.33 (n = 492) SARS-CoV-2 genomes available on GISAID (https://www.gisaid.org/) as of October 19 2020, were downloaded and aligned with Uruguayan and Brazilian sequences of the same lineages generated in this study using MAFFT v7.467[11]. ML phylogenetic trees were constructed under the GTR+F+I+G4 nucleotide substitution model selected by the in-built Smart Model Selection (SMS) option[12] and visualized using FigTree v1.4.4 (http://tree.bio.ed.ac.uk/software/figtree/).

1.6 Bayesian phylogeographic analyses

Temporal signal was assessed by performing a regression analysis of the root-to-tip divergence against sampling time using TempEst[13]. Time-scaled Bayesian trees were estimated using the non-parametric Bayesian skyline (BSKL) model as the coalescent tree prior[14], under the optimal model of nucleotide substitution determined for each data set by the SMS option and applying a strict molecular clock model with a fixed substitution rate (8×10^{-4} substitutions/site/year) based on previous estimates[15,16]. Three MCMC chains were run for 100 and 200 million generations for the B.1.1.28 and B.1.1.33 datasets, respectively, and then combined to ensure stationarity and good mixing. Convergence (Effective Sample Size > 200) in parameter estimates was assessed using TRACER v1.7[17]. The maximum clade credibility (MCC) trees were summarized with TreeAnnotator v1.10 and visualized using FigTree v1.4.4.

1.7 Within-host diversity

In order to study the alternative allele frequencies at the identified synapomorphic sites, base frequencies were directly derived from the adapter trimmed bam files (Tables S4-S7 in section 2). Only those observations where the genome position was covered by at least 100 reads were kept for further analysis. As samples were obtained by three different sequencing technologies (data for 148, 108 and 494 synapomorphic sites was obtained using Illumina, Ion Torrent and ONT, respectively) and sequencing biases exist due to different error profiles, a Shannon Entropy[18] value (H) was estimated per observation based on the four allele frequencies. As diagnostics plots evidence an effect due to the sequencing technology (Figures S1A-B), we determined a linear model to estimate the contributions of sequencing technology, sample and synapomorphic site, to the observed A, C, G, T frequencies (summarized as H): H exp 1⁄4 ~ SEQ + SAMPLE + MUTATION. We used H exp 1⁄4 to better fit a normal distribution (Figure S1C in section 3). This was implemented using the lm function of the R environment [19] . Residuals of the model are shown in Figure S1D (section 3) and coefficients for the covariables are shown in Table S8 (section 2).

The analytical validity of ONT sequencing for rapid SARS-CoV-2 genome analysis has been assessed before and, given a minimum 60x genome coverage, ONT sequencing enables detection of within-specimen single nucleotide variants starting at frequencies ~40% with adequate accuracy (Bull et al, 2020). However, the behavior of IonTorrent sequencing for SARS-CoV-2 genomics is less understood. Given that for six samples of the clade BR-UY-II_{33} (RIV-M5, RIV-M11, RIV-M12, RIV-M13, RIV-M14

and RIV-M15), Illumina, IonTorrent and ONT reads were available, we estimated allele frequencies and entropy H for each sequencing technology. A Spearman rho correlation matrix with associated p-values between estimated entropy variables was obtained with the PerformanceAnalytics R package. Allele frequencies were used to visualize noise levels for calculated alternative frequencies due to sequencing technology. Of note, while available IonTorrent and ONT sequencing data were obtained following the Artic amplicon designs described in the manuscript, Illumina data were obtained through a metagenomic protocol in the context of another study.

Finally, no synapomorphic site in our study is coincident with SARS-CoV-2 genomic sites considered as problematic and recommended to be masked, as can be checked in https://github.com/W-L/ProblematicSites_SARS-CoV2/blob/master/subset_vcf/problematic_sites_sarsCov2.mask.vcf as discussed in https://virological.org/t/issues-with-sars-cov-2-sequencing-data/473/12.

Section 2. Supplementary tables

Table S1: Detailed information of each genome generated for this study by the different sequencing centers. Travel "yes" means that person had recently traveled or was in contact with a recent traveler. Superscript B means that the demultiplexing step was done requiring both barcodes at both ends with min_score above 50 and 40, respectively. Superscript R corresponds to the demultiplexing step without requiring the presence of both barcodes but using min_score above 60.

**Diagnostic Center**	**Sequencing Center**	**Accession ID**	**CollectionDate**	**CollectionSite**	**Sequencing Method**	**Protocol**	**Travel**
SASA	Institut Pasteur de Montevideo	ART-M107	2020-07-08	Artigas	MinION	V3 short amplicons^{R}	
CENUR Regional Norte	Institut Pasteur de Montevideo	ART-M146	2020-07-06	Artigas	MinION	V3 short amplicons^{R}	yes
CENUR Regional Norte	Institut Pasteur de Montevideo	BUN-M151	2020-07-20	Bella Unión	MinION	long amplicons^{R}	
CENUR Regional Norte	Institut Pasteur de Montevideo	BUN-M152	2020-07-22	Bella Unión	MinION	long amplicons^{R}	
CENUR Regional Norte	Institut Pasteur de Montevideo	BUN-M163	2020-07-26	Bella Unión	MinION	V3 short amplicons^{R}	
SASA	Institut Pasteur de Montevideo	CEL-M116	2020-07-17	Río Branco	MinION	long amplicons^{R}	
CURE, Regional Este	Institut Pasteur de Montevideo	CHY-M100	2020-07-16	Chuy	MinION	V3 short amplicons^{R}	
CENUR Regional Norte	IIBCE	RIV-M10	2020-05-21	Rivera	Ion Torrent	V3 short amplicons	
CENUR Regional Norte	IIBCE	RIV-M11	2020-05-21	Rivera	Ion Torrent	V3 short amplicons	
SASA	Institut Pasteur de Montevideo	RIV-M119	2020-07-19	Rivera	MinION	long amplicons^{R}	
CENUR Regional Norte	IIBCE	RIV-M12	2020-05-21	Rivera	Ion Torrent	V3 short amplicons	
CENUR Regional Norte	IIBCE	RIV-M13	2020-05-21	Rivera	Ion Torrent	V3 short amplicons	
CENUR Regional Norte	IIBCE	RIV-M14	2020-05-21	Rivera	Ion Torrent	V3 short amplicons	
CENUR	IIBCE	RIV-M15	2020-05-21	Rivera	Ion Torrent	V3 short	

Regional Norte						amplicons	
CENUR Regional Norte	Institut Pasteur de Montevideo	RIV-M154	2020-07-21	Rivera	MinION	long amplicons[R]	
CENUR Regional Norte	Institut Pasteur de Montevideo	RIV-M155	2020-07-21	Rivera	MinION	V3 short amplicons[R]	
Campus Tacuarembó	Institut Pasteur de Montevideo	RIV-M170	2020-07-20	Rivera	MinION	long amplicons[R]	
SASA	Institut Pasteur de Montevideo	RIV-M40	2020-05-21	Rivera	MinION	long amplicons[B]	
SASA	Institut Pasteur de Montevideo	RIV-M43	2020-05-28	Rivera	MinION	V3 short amplicons[B]	
CENUR Regional Norte	IIBCE	RIV-M5	2020-05-05	Rivera	Ion Torrent	V3 short amplicons	
Campus Tacuarembó	Institut Pasteur de Montevideo	RIV-M72	2020-06-05	Rivera	MinION	V3 short amplicons[B]	
Campus Tacuarembó	Institut Pasteur de Montevideo	RIV-M73	2020-06-05	Rivera	MinION	V3 short amplicons[B]	
CENUR Regional Norte	Institut Pasteur de Montevideo	RIV-M79	2020-06-02	Rivera	MinION	V3 short amplicons[B]	
CENUR Regional Norte	IIBCE	RIV-M8	2020-05-21	Rivera	Ion Torrent	V3 short amplicons	
CENUR Regional Norte	IIBCE	RIV-M9	2020-05-21	Rivera	Ion Torrent	V3 short amplicons	
CENUR Regional Norte	Institut Pasteur de Montevideo	ART-M157	2020-07-23	Artigas	MinION	V3 short amplicons[R]	
SASA	Institut Pasteur de Montevideo	TYT-M130	2020-06-30	Treinta y Tres	MinION	V3 short amplicons[R]	
SASA	Institut Pasteur de Montevideo	TYT-M132	2020-07-01	Treinta y Tres	MinION	V3 short amplicons[R]	
SASA	Institut Pasteur de Montevideo	TYT-M133	2020-07-01	Treinta y Tres	MinION	long amplicons[R]	
SASA	Institut Pasteur de Montevideo	TYT-M135	2020-07-02	Treinta y Tres	MinION	long amplicons[R]	

SASA	Institut Pasteur de Montevideo	TYT-M136	2020-07-02	Treinta y Tres	MinION	long amplicons[R]	
SASA	Institut Pasteur de Montevideo	TYT-M49	2020-06-18	Treinta y Tres	MinION	long amplicons[B]	yes
SASA	Institut Pasteur de Montevideo	TYT-M52	2020-06-18	Treinta y Tres	MinION	V3 short amplicons[R]	
SASA	Institut Pasteur de Montevideo	TYT-M54	2020-06-18	Treinta y Tres	MinION	V3 short amplicons[R]	
SASA	Institut Pasteur de Montevideo	TYT-M57	2020-06-18	Treinta y Tres	MinION	long amplicons[B]	
SASA	Institut Pasteur de Montevideo	TYT-M60	2020-06-19	Treinta y Tres	MinION	V3 short amplicons[R]	
SASA	Institut Pasteur de Montevideo	TYT-M62	2020-06-20	Treinta y Tres	MinION	V3 short amplicons[R]	
SASA	Institut Pasteur de Montevideo	TYT-M63	2020-06-20	Treinta y Tres	MinION	V3 short amplicons[R]	
SASA	Institut Pasteur de Montevideo	TYT-M64	2020-06-21	Treinta y Tres	MinION	long amplicons[B]	
SASA	Institut Pasteur de Montevideo	TYT-M65	2020-06-21	Treinta y Tres	MinION	V3 short amplicons[R]	
SASA	Institut Pasteur de Montevideo	TYT-M66	2020-06-21	Treinta y Tres	MinION	V3 short amplicons[R]	
SASA	Institut Pasteur de Montevideo	TYT-M67	2020-06-21	Treinta y Tres	MinION	V3 short amplicons[R]	
SASA	Institut Pasteur de Montevideo	TYT-M68	2020-06-21	Treinta y Tres	MinION	V3 short amplicons[R]	
SASA	Institut Pasteur de Montevideo	TYT-M69	2020-06-21	Treinta y Tres	MinION	V3 short amplicons[R]	
SASA	Institut Pasteur de Montevideo	TYT-M70	2020-06-21	Treinta y Tres	MinION	V3 short amplicons[R]	
SASA	Institut Pasteur de Montevideo	TYT-M71	2020-06-22	Treinta y Tres	MinION	V3 short amplicons[R]	
SASA	Institut Pasteur de Montevideo	TYT-M85	2020-06-21	Treinta y Tres	MinION	V3 short amplicons[B]	
SASA	Institut Pasteur de Montevideo	TYT-M87	2020-06-24	Treinta y Tres	MinION	V3 short amplicons[B]	

SASA	Institut Pasteur de Montevideo	TYT-M91	2020-06-25	Treinta y Tres	MinION	long amplicons[B]	
SASA	Institut Pasteur de Montevideo	TYT-M93	2020-06-25	Treinta y Tres	MinION	V3 short amplicons[B]	
SASA	Institut Pasteur de Montevideo	TYT-M94	2020-06-26	Treinta y Tres	MinION	V3 short amplicons[B]	
SASA	Institut Pasteur de Montevideo	TYT-M95	2020-06-26	Treinta y Tres	MinION	V3 short amplicons[B]	
SASA	Institut Pasteur de Montevideo	TYT-M97	2020-06-27	Treinta y Tres	MinION	long amplicons[B]	
SASA	Institut Pasteur de Montevideo	TYT-M98	2020-06-27	Treinta y Tres	MinION	V3 short amplicons[B]	
LACEN/RS	LRN, IOC, Fiocruz	hCoV-19/Brazil/RS-15270/2020	13/07/2020	PASSO FUNDO	Illumina Miseq	long amplicons	
LACEN/RS	LRN, IOC, Fiocruz	hCoV-19/Brazil/RS-15273/2020	17/07/2020	CACHOEIRA DO SUL	Illumina Miseq	long amplicons	
LACEN/RS	LRN, IOC, Fiocruz	hCoV-19/Brazil/RS-15279/2020	28/07/2020	NOVA BRESCIA	Illumina Miseq	long amplicons	
LACEN/RS	LRN, IOC, Fiocruz	hCoV-19/Brazil/RS-15283/2020	09/08/2020	PORTO ALEGRE	Illumina Miseq	long amplicons	
LACEN/RS	LRN, IOC, Fiocruz	hCoV-19/Brazil/RS-15286/2020	14/08/2020	SAO BORJA	Illumina Miseq	long amplicons	
LACEN/RS	LRN, IOC, Fiocruz	hCoV-19/Brazil/RS-2525/2020	09/03/2020	PORTO ALEGRE	Illumina Miseq	long amplicons	
LACEN/RS	LRN, IOC, Fiocruz	hCoV-19/Brazil/RS-2528/2020	17/03/2020	ERECHIM	Illumina Miseq	long amplicons	
LACEN/RS	LRN, IOC, Fiocruz	hCoV-19/Brazil/RS-2529/2020	17/03/2020	SANTANA DO LIVRAMENTO	Illumina Miseq	long amplicons	
LACEN/RS	LRN, IOC, Fiocruz	hCoV-19/Brazil/RS-2533/2020	18/03/2020	TORRES	Illumina Miseq	long amplicons	
LACEN/RS	LRN, IOC, Fiocruz	hCoV-19/Brazil/RS-2539/2020	19/03/2020	SANTA MARIA	Illumina Miseq	long amplicons	
LACEN/RS	LRN, IOC, Fiocruz	hCoV-19/Brazil/RS-2544/2020	19/03/2020	TORRES	Illumina Miseq	long amplicons	
LACEN/RS	LRN, IOC,	hCoV-	20/03/2020	PORTO	Illumina	long	

	Fiocruz	19/Brazil/RS-2546/2020		ALEGRE	Miseq	amplicons	
LACEN/RS	LRN, IOC, Fiocruz	hCoV-19/Brazil/RS-2549/2020	22/03/2020	MARAU	Illumina Miseq	long amplicons	
LACEN/RS	LRN, IOC, Fiocruz	hCoV-19/Brazil/RS-2550/2020	20/03/2020	SÃO LEOPOLDO	Illumina Miseq	long amplicons	
LACEN/RS	LRN, IOC, Fiocruz	hCoV-19/Brazil/RS-2553/2020	25/03/2020	SÃO DOMINGOS DO SUL	Illumina Miseq	long amplicons	
LACEN/RS	LRN, IOC, Fiocruz	hCoV-19/Brazil/RS-2554/2020	25/03/2020	PORTO ALEGRE	Illumina Miseq	long amplicons	
LACEN/RS	LRN, IOC, Fiocruz	hCoV-19/Brazil/RS-2556/2020	27/03/2020	FARROUPILHA	Illumina Miseq	long amplicons	
LACEN/RS	LRN, IOC, Fiocruz	hCoV-19/Brazil/RS-2564/2020	03/04/2020	PORTO ALEGRE	Illumina Miseq	long amplicons	
LACEN/RS	LRN, IOC, Fiocruz	hCoV-19/Brazil/RS-2565/2020	03/04/2020	PORTO ALEGRE	Illumina Miseq	long amplicons	
LACEN/RS	LRN, IOC, Fiocruz	hCoV-19/Brazil/RS-2567/2020	06/04/2020	CIDREIRA	Illumina Miseq	long amplicons	
LACEN/RS	LRN, IOC, Fiocruz	hCoV-19/Brazil/RS-6169/2020	12/03/2020	PASSO FUNDO	Illumina Miseq	long amplicons	
LACEN/RS	LRN, IOC, Fiocruz	hCoV-19/Brazil/RS-6177/2020	06/05/2020	CARLOS BARBOSA	Illumina Miseq	long amplicons	
LACEN/RS	LRN, IOC, Fiocruz	hCoV-19/Brazil/RS-6179/2020	06/05/2020	SANTA ROSA	Illumina Miseq	long amplicons	
LACEN/RS	LRN, IOC, Fiocruz	hCoV-19/Brazil/RS-6180/2020	06/05/2020	VENÂNCIO AIRES	Illumina Miseq	long amplicons	
LACEN/RS	LRN, IOC, Fiocruz	hCoV-19/Brazil/RS-6183/2020	06/05/2020	SALDANHA MARINHO	Illumina Miseq	long amplicons	
LACEN/RS	LRN, IOC, Fiocruz	hCoV-19/Brazil/RS-6184/2020	08/05/2020	ITAQUI	Illumina Miseq	long amplicons	
LACEN/RS	LRN, IOC, Fiocruz	hCoV-19/Brazil/RS-6187/2020	09/05/2020	VILA MARIA	Illumina Miseq	long amplicons	
LACEN/RS	LRN, IOC,	hCoV-	10/05/2020	CAXIAS DO	Illumina	long	

	Fiocruz	19/Brazil/RS-6188/2020		SUL	Miseq	amplicons	
LACEN/RS	LRN, IOC, Fiocruz	hCoV-19/Brazil/RS-6189/2020	10/05/2020	CAXIAS DO SUL	Illumina Miseq	long amplicons	
LACEN/RS	LRN, IOC, Fiocruz	hCoV-19/Brazil/RS-6190/2020	11/05/2020	ARROIO DO MEIO	Illumina Miseq	long amplicons	
LACEN/RS	LRN, IOC, Fiocruz	hCoV-19/Brazil/RS-6192/2020	12/05/2020	SERAFINA CORREA	Illumina Miseq	long amplicons	
LACEN/RS	LRN, IOC, Fiocruz	hCoV-19/Brazil/RS-6195/2020	11/05/2020	NOVA ARAÇA	Illumina Miseq	long amplicons	
LACEN/RS	LRN, IOC, Fiocruz	hCoV-19/Brazil/RS-6196/2020	12/05/2020	PASSO FUNDO	Illumina Miseq	long amplicons	
LACEN/RS	LRN, IOC, Fiocruz	hCoV-19/Brazil/RS-6197/2020	13/05/2020	NÃO ME TOQUE	Illumina Miseq	long amplicons	
LACEN/RS	LRN, IOC, Fiocruz	hCoV-19/Brazil/RS-6198/2020	14/05/2020	SERAFINA CORREA	Illumina Miseq	long amplicons	
LACEN/RS	LRN, IOC, Fiocruz	hCoV-19/Brazil/RS-6203/2020	14/05/2020	QUARAÍ	Illumina Miseq	long amplicons	
LACEN/RS	LRN, IOC, Fiocruz	hCoV-19/Brazil/RS-6205/2020	18/05/2020	SANTANA DO LIVRAMENTO	Illumina Miseq	long amplicons	
LACEN/RS	LRN, IOC, Fiocruz	hCoV-19/Brazil/RS-6208/2020	19/05/2020	FREDERICO WESTPHALEN	Illumina Miseq	long amplicons	
LACEN/RS	LRN, IOC, Fiocruz	hCoV-19/Brazil/RS-6213/2020	20/04/2020	PASSO FUNDO	Illumina Miseq	long amplicons	
LACEN/RS	LRN, IOC, Fiocruz	hCoV-19/Brazil/RS-6215/2020	20/05/2020	PASSO FUNDO	Illumina Miseq	long amplicons	
LACEN/RS	LRN, IOC, Fiocruz	hCoV-19/Brazil/RS-6218/2020	27/05/2020	VAIMAO	Illumina Miseq	long amplicons	
LACEN/RS	LRN, IOC, Fiocruz	hCoV-19/Brazil/RS-6219/2020	27/05/2020	PORTO ALEGRE	Illumina Miseq	long amplicons	
LACEN/RS	LRN, IOC, Fiocruz	hCoV-19/Brazil/RS-6220/2020	27/05/2020	PORTO ALEGRE	Illumina Miseq	long amplicons	
LACEN/RS	LRN, IOC, Fiocruz	hCoV-19/Brazil/RS-6222/2020	28/05/2020	FLORES DA CUNHA	Illumina Miseq	long amplicons	
LACEN/RS	LRN, IOC, Fiocruz	hCoV-19/Brazil/RS-	29/05/2020	VENANCIO AIRES	Illumina Miseq	long amplicons	

		6227/2020					
LACEN/RS	LRN, IOC, Fiocruz	hCoV-19/Brazil/RS-6228/2020	28/05/2020	LAJEADO	Illumina Miseq	long amplicons	
LACEN/RS	LRN, IOC, Fiocruz	hCoV-19/Brazil/RS-6231/2020	29/05/2020	CAÇAPAVA DO SUL	Illumina Miseq	long amplicons	
LACEN/RS	LRN, IOC, Fiocruz	hCoV-19/Brazil/RS-6232/2020	28/05/2020	BAGE	Illumina Miseq	long amplicons	
LACEN/RS	LRN, IOC, Fiocruz	hCoV-19/Brazil/RS-6240/2020	01/06/2020	SANTO ANGELO	Illumina Miseq	long amplicons	
LACEN/RS	LRN, IOC, Fiocruz	hCoV-19/Brazil/RS-6241/2020	01/06/2020	GIRUA	Illumina Miseq	long amplicons	
LACEN/RS	LRN, IOC, Fiocruz	hCoV-19/Brazil/RS-6242/2020	31/05/2020	CRUZ ALTA	Illumina Miseq	long amplicons	
LACEN/RS	LRN, IOC, Fiocruz	hCoV-19/Brazil/RS-6226/2020	29/05/2020	BARRA DO RIBEIRO	IonTorrent	long amplicons	
LACEN/RS	LRN, IOC, Fiocruz	hCoV-19/Brazil/RS-6243/2020	02/06/2020	IGREJINHA	IonTorrent	long amplicons	
LACEN/RS	LRN, IOC, Fiocruz	hCoV-19/Brazil/RS-15274/2020	17/07/2020	PORTO ALEGRE	Nanopore MinION	long amplicons	
LACEN/RS	LRN, IOC, Fiocruz	hCoV-19/Brazil/RS-15275/2020	18/07/2020	ARROIO GRANDE	Nanopore MinION	long amplicons	
LACEN/RS	LRN, IOC, Fiocruz	hCoV-19/Brazil/RS-15276/2020	20/07/2020	BOM RETIRO DO SUL	Nanopore MinION	long amplicons	
LACEN/RS	LRN, IOC, Fiocruz	hCoV-19/Brazil/RS-15278/2020	27/07/2020	TAPEJARA	Nanopore MinION	long amplicons	
LACEN/RS	LRN, IOC, Fiocruz	hCoV-19/Brazil/RS-15280/2020	28/07/2020	PORTO ALEGRE	Nanopore MinION	long amplicons	
LACEN/RS	LRN, IOC, Fiocruz	hCoV-19/Brazil/RS-15281/2020	07/08/2020	FARROUPILHA	Nanopore MinION	long amplicons	
LACEN/RS	LRN, IOC, Fiocruz	hCoV-19/Brazil/RS-15282/2020	06/08/2020	CAXIAS DO SUL	Nanopore MinION	long amplicons	
LACEN/RS	LRN, IOC, Fiocruz	hCoV-19/Brazil/RS-15284/2020	12/08/2020	IJUI	Nanopore MinION	long amplicons	
LACEN/RS	LRN, IOC, Fiocruz	hCoV-19/Brazil/RS-15285/2020	14/08/2020	VENANCIO AIRES	Nanopore MinION	long amplicons	
LACEN/RS	LRN, IOC,	hCoV-	13/08/2020	SANTA ROSA	Nanopore	long	

	Fiocruz	19/Brazil/RS-15287/2020			MinION	amplicons	
LACEN/RS	LRN, IOC, Fiocruz	hCoV-19/Brazil/RS-15288/2020	12/08/2020	NAO-ME-TOQUE	Nanopore MinION	long amplicons	
LACEN/RS	LRN, IOC, Fiocruz	hCoV-19/Brazil/RS-15289/2020	14/08/2020	PORTO ALEGRE	Nanopore MinION	long amplicons	
LACEN/RS	LRN, IOC, Fiocruz	hCoV-19/Brazil/RS-15290/2020	15/08/2020	OSORIO	Nanopore MinION	long amplicons	
LACEN/RS	LRN, IOC, Fiocruz	hCoV-19/Brazil/RS-15291/2020	15/08/2020	TRES PASSOS	Nanopore MinION	long amplicons	
LACEN/RS	LRN, IOC, Fiocruz	hCoV-19/Brazil/RS-15292/2020	16/08/2020	PORTO ALEGRE	Nanopore MinION	long amplicons	

Table S2. Accession IDs Uruguayan Genomes

Accession ID
EPI_ISL_747615
EPI_ISL_749238
EPI_ISL_748667
EPI_ISL_748143
EPI_ISL_748142
EPI_ISL_749036
EPI_ISL_748141
EPI_ISL_748140
EPI_ISL_749152
EPI_ISL_749153
EPI_ISL_748145
EPI_ISL_749154
EPI_ISL_748144
EPI_ISL_749155
EPI_ISL_749474
EPI_ISL_749150
EPI_ISL_749151
EPI_ISL_750108
EPI_ISL_750430
EPI_ISL_750820
EPI_ISL_749906
EPI_ISL_748139
EPI_ISL_748138
EPI_ISL_749706
EPI_ISL_749149

EPI_ISL_749148
EPI_ISL_750167
EPI_ISL_750165
EPI_ISL_750166
EPI_ISL_750163
EPI_ISL_750164
EPI_ISL_751011
EPI_ISL_750161
EPI_ISL_750162
EPI_ISL_750169
EPI_ISL_750170
EPI_ISL_750171
EPI_ISL_750178
EPI_ISL_750179
EPI_ISL_750256
EPI_ISL_750176
EPI_ISL_750177
EPI_ISL_750174
EPI_ISL_750175
EPI_ISL_750172
EPI_ISL_751184
EPI_ISL_750173
EPI_ISL_751186
EPI_ISL_751187
EPI_ISL_751185
EPI_ISL_751190
EPI_ISL_751201
EPI_ISL_751188
EPI_ISL_751189

Table S9. GISAID acknowledgement table for B1.1.33 sequences

We gratefully acknowledge the following Authors from the Originating laboratories responsible for obtaining the specimens, as well as the Submitting laboratories where the genome data were generated and shared via GISAID, on which this research is based.

All Submitters of data may be contacted directly via www.gisaid.org

Accession ID	Originating Laboratory	Submitting Laboratory	Authors
EPI_ISL_418371	Public Health Ontario Laboratories	Public Health Ontario Laboratories	Alireza Eshaghi, Samir N Patel, Jonathan B Gubbay, Vanessa G Allen, Christine Frantz, Aimin Li, Sandeep Nagra
EPI_ISL_425171	University of Wisconsin-Madison AIDS Vaccine Research Laboratories	University of Wisconsin-Madison AIDS Vaccine Research Laboratories	Gage Moreno, Katarina Braun, et al. AIDS Vaccine Research Laboratories
EPI_ISL_425373	Department of Pathology, University of Cambridge	COVID-19 Genomics UK (COG-UK) Consortium	Luke W Meredith, M. Estee Torok , Myra Hosmillo, William L. Hamilton, Martin D. Curran, Theresa Feltwell, Anna Yakovleva, Charlotte J. Houldcroft, Aminu S. Jahun, Sarah L. Caddy, Ian Goodfellow
EPI_ISL_426899	Royal Darwin Hospital Pathology	Microbiological Diagnostic Unit Public Health Laboratory and Victorian Infectious Diseases Reference Laboratory, Doherty Institute	Meumann, E., Caly L., Seemann T., Sait, M., Schultz M., Druce J., Sherry, N.
EPI_ISL_427131	Victorian Infectious Diseases Reference Laboratory (VIDRL)	Microbiological Diagnostic Unit Public Health Laboratory and Victorian Infectious Diseases Reference Laboratory, Doherty Institute	Caly L., Seemann T., Sait, M., Schultz M., Druce J., Sherry, N.
EPI_ISL_427294, EPI_ISL_427295, EPI_ISL_427296, EPI_ISL_427297, EPI_ISL_427298, EPI_ISL_427302, EPI_ISL_427303, EPI_ISL_427304	Instituto Oswaldo Cruz FIOCRUZ - Laboratory of Respiratory Viruses and Measles (LVRS)	Instituto Oswaldo Cruz FIOCRUZ - Laboratory of Respiratory Viruses and Measles (LVRS)	Paola Resende, Fernando Motta, Luciana Appolinario, Sunando Roy, Aline Mattos, Milene Miranda, Cristiana Garcia, Braulia Caetano, Maria Ogrzewalska, Priscila Born, Jonathan Lopes, Marilda Siqueira
EPI_ISL_428294	University of Wisconsin-Madison AIDS Vaccine Research Laboratories	University of Wisconsin-Madison AIDS Vaccine Research Laboratories	Gage Moreno, Katarina Braun, et al. AIDS Vaccine Research Laboratories
EPI_ISL_430814, EPI_ISL_430815, EPI_ISL_430817	Laboratorio de Virología del Hospital de Niños Dr. Ricardo Gutierrez	Área de Secuenciación del Laboratorio de Virología del Hospital de Niños Dr. Ricardo Gutierrez	Nabaes Jodar, MS; Goya, S; Natale, MI; Lusso, S; Gravis, E; Mistchenko, AS; Valinotto, LE; Viegas, M.
EPI_ISL_434803	Houston Methodist Hospital	Houston Methodist Hospital	S. Wesley Long, Randall J. Olsen, Paul A. Christensen, David W. Bernard, James J. Davis, Maulik Shukla, Marcus Nguyen, Matthew Ojeda Saavedra, Concepcion C. Cantu, Prasanti Yerramilli, Layne Pruitt, Sishir Subedi, Heather Hendrickson, Ghazaleh Eskandari, Muthiah Kumaraswami, Jason S. McLellan, Hakon Jonsson, Kari Stefansson, and James M. Musser
EPI_ISL_436853, EPI_ISL_437100	Michigan Department of Health and Human Services, Bureau of Laboratories	Michigan Department of Health and Human Services, Bureau of Laboratories	Blankenship HM, Riner D, Soehnlen MK
EPI_ISL_437806	UW Virology Lab	UW Virology Lab	Pavitra Roychoudhury, Hong Xie, Keith Jerome, Alexander Greninger
EPI_ISL_444626	NYU Langone Health	Departments of Pathology and Medicine, New York University School of Medicine	Maria Aguero-Rosenfeld, Brendan Belovarac, Margaret Black, Ludovic Boytard, John Cadley, Paolo Cotzia, John Chen, Dacia Dimartino, Xiaojun Feng, Tatyana Gindin, Emily Guzman, Adriana Heguy, Megan Hogan, Emily Huang, George Jour, Alireza Khodadadi-Jamayran, Lawrence H. Lin, Raven Luther, Andrew Lytle, Christian Marier, Matthew T. Maurano, Mark J. Mulligan, Peter Meyn, Raquel Ordonez Ciriza, Iman Osman, Jared Pinnell, Vanessa Raabe, Sitharam Ramaswami, Amy Rapkiewicz, Andre M. Ribeiro-dos-Santos, Marie Samanovic-Golden, Antonio Serrano, Guomiao Shen, Matija Snuderl, Theodore Vougiouklakis, Nick Vulpescu, Gael Westby, Paul Zappile, Yutong Zhang
EPI_ISL_445349	HOSPITAL SAN JUAN DE DIOS	Instituto de Salud Publica de Chile	Andrés E Castillo, Bárbara Parra,Paz Tapia, Jaime Lagos, Loredana Arata, Alejandra Acevedo, Winston Andrade, Gabriel Leal, Carolina Tambley, Patricia Bustos, Rodrigo Fasce, Jorge Fernandez
EPI_ISL_445352	HOSPITAL DEL PROFESOR	Instituto de Salud Publica de Chile	Andrés E Castillo, Bárbara Parra,Paz Tapia, Jaime Lagos, Loredana Arata, Alejandra Acevedo, Winston Andrade, Gabriel Leal, Carolina Tambley, Patricia Bustos, Rodrigo Fasce, Jorge Fernandez
EPI_ISL_445362	BUPA SERVICIOS CLINICOS S.A	Instituto de Salud Publica de Chile	Andrés E Castillo, Bárbara Parra,Paz Tapia, Jaime Lagos, Loredana Arata, Alejandra Acevedo, Winston Andrade, Gabriel Leal, Carolina Tambley, Patricia Bustos, Rodrigo Fasce, Jorge Fernandez
EPI_ISL_445367	ASISTENCIA PUBLICA DR.ALEJANDRO DEL RIO	Instituto de Salud Publica de Chile	Andrés E Castillo, Bárbara Parra,Paz Tapia, Jaime Lagos, Loredana Arata, Alejandra Acevedo, Winston Andrade, Gabriel Leal, Carolina Tambley, Patricia Bustos, Rodrigo Fasce, Jorge Fernandez
EPI_ISL_445369, EPI_ISL_445370	HOSPITAL DE CARABINEROS	Instituto de Salud Publica de Chile	Andrés E Castillo, Bárbara Parra,Paz Tapia, Jaime Lagos, Loredana Arata, Alejandra Acevedo, Winston Andrade, Gabriel Leal, Carolina Tambley, Patricia Bustos, Rodrigo Fasce, Jorge Fernandez
EPI_ISL_445373	HOSPITAL SAN JUAN DE DIOS	Instituto de Salud Publica de Chile	Andrés E Castillo, Bárbara Parra,Paz Tapia, Jaime Lagos, Loredana Arata, Alejandra Acevedo, Winston Andrade, Gabriel Leal, Carolina Tambley, Patricia Bustos, Rodrigo Fasce, Jorge Fernandez
EPI_ISL_450873	Evandro Chagas Institute	Evandro Chagas Institute	Santos, M.C.; Silva, A.M.; Junior, W.D.C.; Barbagelata, L.S.; Ferreira, J.A.; Sousa, E.M.A.; da Silva, P.S.; Martins, L.C.; Sousa Junior, E.C.; Viana, G.M.R
EPI_ISL_450874	Evandro Chagas Institute	Evandro Chagas Institute	Santos, M.C.; Silva, A.M.; Junior, W.D.C.; Barbagelata, L.S.; Ferreira, J.A.; Sousa, E.M.A.; da Silva, P.S.; Martins, L.C.;Sousa Junior, E.C.;Viana, G.M.R
EPI_ISL_451558	Medlab Pathology	NSW Health Pathology - Institute of Clinical Pathology and Medical Research; Westmead Hospital; University of Sydney	CIDM-PH et al.
EPI_ISL_451594	Childrens Hospital Westmead	NSW Health Pathology - Institute of Clinical Pathology and Medical Research; Westmead Hospital; University of Sydney	CIDM-PH et al.
EPI_ISL_452318	Michigan Department of Health and Human Services, Bureau of Laboratories	Michigan Department of Health and Human Services, Bureau of Laboratories	Blankenship HM, Riner D, Soehnlen MK
EPI_ISL_454057, EPI_ISL_454172, EPI_ISL_454311	unknown	Instituto Nacional de Saude (INSA)	Borges et al
EPI_ISL_454376	UPMC Clinical Microbiology Laboratory	Microbial Genome Sequencing Center, Microbial Genomic Epidemiological Laboratory	Mustapha M. Mustapha, Jane W. Marsh, Dan Snyder, Marissa P. Griffith, Stephanie L. Mitchell, Vatsala R. Srinivasa, Kady D. Waggle, Chinelo Ezeonwuku, Vaughn S. Cooper, Lee H. Harrison
EPI_ISL_456071, EPI_ISL_456072, EPI_ISL_456073, EPI_ISL_456074, EPI_ISL_456075	Laboratory of Respiratory Viruses and Measles, Oswaldo Cruz Institute, FIOCRUZ	Laboratory of Respiratory Viruses and Measles, Oswaldo Cruz Institute, FIOCRUZ	Paola Resende, Luciana Appolinario, Fernando Motta, Aline Mattos, Milene Miranda, Cristiana Garcia, Braulia Caetano, Maria Ogrzewalska, Jonathan Lopes, Marilda Siqueira
EPI_ISL_456076, EPI_ISL_456077	LACEN RJ - Laboratório Central de Saúde Pública Noel Nutels	Laboratory of Respiratory Viruses and Measles, Oswaldo Cruz Institute, FIOCRUZ	Paola Resende, Luciana Appolinario, Fernando Motta, Aline Mattos, Milene Miranda, Cristiana Garcia, Braulia Caetano, Maria Ogrzewalska, Jonathan Lopes, Marilda Siqueira
EPI_ISL_456079, EPI_ISL_456080, EPI_ISL_456081	Laboratory of Respiratory Viruses and Measles, Oswaldo Cruz Institute, FIOCRUZ	Laboratory of Respiratory Viruses and Measles, Oswaldo Cruz Institute, FIOCRUZ	Paola Resende, Luciana Appolinario, Fernando Motta, Aline Mattos, Milene Miranda, Cristiana Garcia, Braulia Caetano, Maria Ogrzewalska, Jonathan Lopes, Marilda Siqueira
EPI_ISL_456082, EPI_ISL_456083	LACEN RJ - Laboratório Central de Saúde Pública Noel Nutels	Laboratory of Respiratory Viruses and Measles, Oswaldo Cruz Institute, FIOCRUZ	Paola Resende, Luciana Appolinario, Fernando Motta, Aline Mattos, Milene Miranda, Cristiana Garcia, Braulia Caetano, Maria Ogrzewalska, Jonathan Lopes, Marilda Siqueira
EPI_ISL_456084, EPI_ISL_456085, EPI_ISL_456086, EPI_ISL_456087, EPI_ISL_456089, EPI_ISL_456090, EPI_ISL_456091, EPI_ISL_456092, EPI_ISL_456093, EPI_ISL_456094, EPI_ISL_456095, EPI_ISL_456096, EPI_ISL_456097, EPI_ISL_456098, EPI_ISL_456099, EPI_ISL_456100, EPI_ISL_456101, EPI_ISL_456102, EPI_ISL_456103, EPI_ISL_456104, EPI_ISL_456105, EPI_ISL_456106			
see above	Laboratory of Respiratory Viruses and Measles, Oswaldo Cruz Institute, FIOCRUZ	Laboratory of Respiratory Viruses and Measles, Oswaldo Cruz Institute, FIOCRUZ	Paola Resende, Luciana Appolinario, Fernando Motta, Aline Mattos, Milene Miranda, Cristiana Garcia, Braulia Caetano, Maria Ogrzewalska, Jonathan Lopes, Marilda Siqueira
EPI_ISL_457796	Johns Hopkins Hospital Department of Pathology	Johns Hopkins Hospital Department of Pathology	Peter M. Thielen, Thomas Mehoke, Shirlee Wohl, Srividya Ramakrishnan, Melanie Kirsche, Amanda Ernlund, Craig Howser, Kristina Zudock, Oluwaseun Falade-Nwulia, Norah Sadowski, Paul Morris, Mark Hopkins, Yunfan Fan, Nidia Trovao, Victoria Gniazdowski, Michael C. Schatz, Stuart C. Ray, Winston Timp, Heba H. Mostafa
EPI_ISL_457953	Laboratorio de Biología Molecular Asociación Española Primera en Salud	Departments of Pathology and Medicine, New York University School of Medicine	Maria Victoria Elizondo, Maria Noel Zubillaga, Gonzalo Manrique, Paul Zappile, Gael Westby, Matthew T Maurano, Christian Marier, Adriana Heguy
EPI_ISL_458138, EPI_ISL_458139, EPI_ISL_458142, EPI_ISL_458143, EPI_ISL_458144, EPI_ISL_458145, EPI_ISL_458148, EPI_ISL_458149	Evandro Chagas Institute	Evandro Chagas Institute	Santos, M.C.; Silva, A.M.; Junior, W.D.C.; Barbagelata, L.S.; Ferreira, J.A.; Sousa, E.M.A.; da Silva, P.S.; Resque, H.R; Martins, L.C.; Sousa Junior, E.C.;Viana, G.M.R
EPI_ISL_460134, EPI_ISL_460202, EPI_ISL_460215, EPI_ISL_460236, EPI_ISL_460403	Massachusetts General Hospital	Infectious Disease Program, Broad Institute of Harvard and MIT	Lemieux,J.E., Siddle,K.J., Shaw,B., Adams,G., Pierce,V., Turbett,S., Anahtar,M., Branda,J., Slater,D., Harris,J., Lin,A.E., Gladden-Young,A., Lagerborg,K., Rudy,M., DeRuff,K., Carter,A., Normandin,E., Bauer,M., Reilly,S., Tomkins-Tinch,C., Loreth,C., Chaluvadi,S., Neumann,A., Cusick,C., Chapman,S.B., Gnirke,A., Flowers,K., Cerrato,F., Birren,B.W., Gallagher,G., Smole,S., Park,D.J., MacInnis,B.L., Ryan,E., LaRocque,R., Rosenberg,E., Sabeti,P.C.
EPI_ISL_465088	Respiratory Virus Unit, Microbiology Services Colindale, Public Health England	Respiratory Virus Unit, Microbiology Services Colindale, Public Health England	PHE Covid Sequencing Team
EPI_ISL_467345, EPI_ISL_467347, EPI_ISL_467348, EPI_ISL_467349, EPI_ISL_467350, EPI_ISL_467351, EPI_ISL_467352, EPI_ISL_467353, EPI_ISL_467355, EPI_ISL_467357, EPI_ISL_467358, EPI_ISL_467360, EPI_ISL_467361, EPI_ISL_467362, EPI_ISL_467363, EPI_ISL_467364, EPI_ISL_467365, EPI_ISL_467367, EPI_ISL_467368, EPI_ISL_467369, EPI_ISL_467370, EPI_ISL_467371			
see above	Laboratory of Respiratory Viruses and Measles, Oswaldo Cruz Institute, FIOCRUZ	Laboratory of Respiratory Viruses and Measles, Oswaldo Cruz Institute, FIOCRUZ	Paola Resende, Luciana Appolinario, Fernando Motta, Anna Carolina Paixão, Ana Carolina Mendonça, Aline Mattos, Milene Miranda, Cristiana Garcia, Braulia Caetano, Maria Ogrzewalska, Jonathan Lopes, Marilda Siqueira

Table S10. GISAID acknowledgement table for B1.1.28 sequences

We gratefully acknowledge the following Authors from the Originating laboratories responsible for obtaining the specimens, as well as the Submitting laboratories where the genome data were generated and shared via GISAID, on which this research is based.

All Submitters of data may be contacted directly via www.gisaid.org

Accession ID	Originating Laboratory	Submitting Laboratory	Authors
EPI_ISL_416036	National Influenza Center - Instituto Adolfo Lutz	Instituto Adolfo Lutz, Interdiciplinary Procedures Center, Strategic Laboratory	Claudio Tavares Sacchi, Claudia Regina Gonçalves, Carlos Henrique Camargo, Erica Valessa Ramos Gomes, Fabiana Cristina Pereira dos Santos, Daniela Bernardes Borges da Silva, Simone Guadagnucci Morillo, Adriano Abbud, Adriana Bugno, Maria do Carmo Sampaio Tavares Timenetsky, Terezinha Maria de Paiva
EPI_ISL_427292	LACEN-AL - Laboratorio Central de Alagoas	Instituto Oswaldo Cruz FIOCRUZ - Laboratory of Respiratory Viruses and Measles (LVRS)	Paola Resende, Fernando Motta, Luciana Appolinario, Sunando Roy, Aline Mattos, Milene Miranda, Cristiana Garcia, Braulia Caetano, Maria Ogrzewalska, Priscila Born, Jonathan Lopes, Marilda Siqueira
EPI_ISL_431180, EPI_ISL_431240	Fujian Center for Disease Control and Prevention	Fujian Center for Disease Control and Prevention	Lin Qi, Huang Zhimiao, Zhang Yanhua, Weng Yuwei
EPI_ISL_445380	Ramathibodi Hospital	COVID-19 Network Investigations (CONI) Alliance	Elizabeth Batty, Wasun Chantratita, Thanat Chookajorn, Stefan Fernandez, Angkana Huang, Anthony R. Jones, Khajohn Joonsalak, Chonticha Klungtong, Theerarat Kochakarn, Namfon Kotanan, Krittikorn Kumpornsin, Wudtichai Manasatienkij, Bhakbhoom Panthan, Ekawat Pasomsub, Kingkan Rakmanee, Insee Sensorn, Janjira Thaipadungpanit, Arporn Wangwiwatsin, Treewat Watthanachockchai
EPI_ISL_456088	LACEN RJ - Laboratório Central de Saúde Pública Noel Nutels	Laboratory of Respiratory Viruses and Measles, Oswaldo Cruz Institute, FIOCRUZ	Paola Resende, Luciana Appolinario, Fernando Motta, Aline Mattos, Milene Miranda, Cristiana Garcia, Braulia Caetano, Maria Ogrzewalska, Jonathan Lopes, Marilda Siqueira
EPI_ISL_456869	West of Scotland Specialist Virology Centre, NHSGGC / MRC-University of Glasgow Centre for Virus Research	COVID-19 Genomics UK (COG-UK) Consortium	Ana da Silva Filipe, Natasha Johnson, Kathy Smollett, Daniel Mair, Stephen Carmichael, Lily Tong, Jenna Nichols, Elihu Aranday-Cortes, Kirstyn Brunker, Yasmin Parr, Kyriaki Nomikou; Sarah McDonald, Marc Niebel, Patawee Asamaphan; Richard Orton, Joseph Hughes, Sreenu Vattipally, David L Robertson; Alasdair MacLean, Rory Gunson; Kathy Li, Natasha Jesudason, Rajiv Shah, James Shepherd, Antonia Ho, Emma Thomson
EPI_ISL_458140, EPI_ISL_458141, EPI_ISL_458146, EPI_ISL_458147	Evandro Chagas Institute	Evandro Chagas Institute	Santos, M.C.; Silva, A.M.; Junior, W.D.C.; Barbagelata, L.S.; Ferreira, J.A.; Sousa, E.M.A.; da Silva, P.S.; Resque, H.R; Martins, L.C.; Sousa Junior, E.C.;Viana, G.M.R
EPI_ISL_461606, EPI_ISL_461678	West of Scotland Specialist Virology Centre, NHSGGC / MRC-University of Glasgow Centre for Virus Research	COVID-19 Genomics UK (COG-UK) Consortium	Ana da Silva Filipe, Natasha Johnson, Kathy Smollett, Daniel Mair, Stephen Carmichael, Lily Tong, Jenna Nichols, Elihu Aranday-Cortes, Kirstyn Brunker, Yasmin Parr, Kyriaki Nomikou; Sarah McDonald, Marc Niebel, Patawee Asamaphan; Richard Orton, Joseph Hughes, Sreenu Vattipally, David L Robertson; Alasdair MacLean, Rory Gunson; Kathy Li, Natasha Jesudason, Rajiv Shah, James Shepherd, Antonia Ho, Emma Thomson
EPI_ISL_467356, EPI_ISL_467359, EPI_ISL_467366	Laboratory of Respiratory Viruses and Measles, Oswaldo Cruz Institute, FIOCRUZ	Laboratory of Respiratory Viruses and Measles, Oswaldo Cruz Institute, FIOCRUZ	Paola Resende, Luciana Appolinario, Fernando Motta, Anna Carolina Paixão, Ana Carolina Mendonça, Aline Mattos, Milene Miranda, Cristiana Garcia, Braulia Caetano, Maria Ogrzewalska, Jonathan Lopes, Marilda Siqueira
EPI_ISL_468305, EPI_ISL_468307	Centro de Vigilancia a Saude de Diadema	Instituto Adolfo Lutz, Interdiciplinary Procedures Center, Strategic Laboratory	Claudio Tavares Sacchi, Claudia Regina Gonçalves, Erica Valessa Ramos Gomes
EPI_ISL_468308	Hospital Municipal do Tatuape Carmino Caricchio	Instituto Adolfo Lutz, Interdiciplinary Procedures Center, Strategic Laboratory	Claudio Tavares Sacchi, Claudia Regina Gonçalves, Erica Valessa Ramos Gomes
EPI_ISL_468311, EPI_ISL_468312	Hospital Municipal Dr Ignacio Proenca de Gouvea	Instituto Adolfo Lutz, Interdiciplinary Procedures Center, Strategic Laboratory	Claudio Tavares Sacchi, Claudia Regina Gonçalves, Erica Valessa Ramos Gomes
EPI_ISL_468313	Vigilancia Epidemiologica de São Bernardo do Campo	Instituto Adolfo Lutz, Interdiciplinary Procedures Center, Strategic Laboratory	Claudio Tavares Sacchi, Claudia Regina Gonçalves, Erica Valessa Ramos Gomes
EPI_ISL_468314	CTA Centro de Testagem e Aconselhamento	Instituto Adolfo Lutz, Interdiciplinary Procedures Center, Strategic Laboratory	Claudio Tavares Sacchi, Claudia Regina Gonçalves, Erica Valessa Ramos Gomes
EPI_ISL_468315	Hospital Municipal do Tatuape Carmino Caricchio	Instituto Adolfo Lutz, Interdiciplinary Procedures Center, Strategic Laboratory	Claudio Tavares Sacchi, Claudia Regina Gonçalves, Erica Valessa Ramos Gomes
EPI_ISL_468316	UPA Vila Assis	Instituto Adolfo Lutz, Interdiciplinary Procedures Center, Strategic Laboratory	Claudio Tavares Sacchi, Claudia Regina Gonçalves, Erica Valessa Ramos Gomes
EPI_ISL_468318	Hospital Universitario da USP	Instituto Adolfo Lutz, Interdiciplinary Procedures Center, Strategic Laboratory	Claudio Tavares Sacchi, Claudia Regina Gonçalves, Erica Valessa Ramos Gomes
EPI_ISL_468319	Vigilancia Epidemiologica de São Bernardo do Campo	Instituto Adolfo Lutz, Interdiciplinary Procedures Center, Strategic Laboratory	Claudio Tavares Sacchi, Claudia Regina Gonçalves, Erica Valessa Ramos Gomes
EPI_ISL_468321	Hospital Universitario da USP	Instituto Adolfo Lutz, Interdiciplinary Procedures Center, Strategic Laboratory	Claudio Tavares Sacchi, Claudia Regina Gonçalves, Erica Valessa Ramos Gomes
EPI_ISL_470600, EPI_ISL_470602, EPI_ISL_470604, EPI_ISL_470605, EPI_ISL_470608, EPI_ISL_470610, EPI_ISL_470612	Hermes Pardini	Bioinformatics Laboratory / LNCC	Alexandra Gerber, Ana Paula Guimarães, Luiz Gonzaga Paula de Almeida, Ronaldo da Silva Francisco Junior, Mariane Talon, Filipe Romero, Átila Duque Rossi, Terezinha Marta Pereira, working group UFRJ, Jaqueline Goes de Jesus, Ingra Morales Claro, Ester Cerdeira Sabino, Nuno Rodrigues Faria, CADDE-group, Laboratorio Hermes Pardini, Laboratorio Simile, working group UFMG, Amilcar Tanuri, Carolina Voloch, Renato Santana Aguiar e Ana Tereza Vasconcelos
EPI_ISL_470638	Laboratorio de Virologia Molecular / UFRJ	Bioinformatics Laboratory / LNCC	Alexandra Gerber, Ana Paula Guimarães, Luiz Gonzaga Paula de Almeida, Ronaldo da Silva Francisco Junior, Mariane Talon, Filipe Romero, Átila Duque Rossi, Terezinha Marta Pereira, working group UFRJ, Jaqueline Goes de Jesus, Ingra Morales Claro, Ester Cerdeira Sabino, Nuno Rodrigues Faria, CADDE-group, Laboratorio Hermes Pardini, Laboratorio Simile, working group UFMG, Amilcar Tanuri, Carolina Voloch, Renato Santana Aguiar e Ana Tereza Vasconcelos
EPI_ISL_470651, EPI_ISL_470653, EPI_ISL_470654	Hermes Pardini	Bioinformatics Laboratory / LNCC	Alexandra Gerber, Ana Paula Guimarães, Luiz Gonzaga Paula de Almeida, Ronaldo da Silva Francisco Junior, Mariane Talon, Filipe Romero, Átila Duque Rossi, Terezinha Marta Pereira, working group UFRJ, Jaqueline Goes de Jesus, Ingra Morales Claro, Ester Cerdeira Sabino, Nuno Rodrigues Faria, CADDE-group, Laboratorio Hermes Pardini, Laboratorio Simile, working group UFMG, Amilcar Tanuri, Carolina Voloch, Renato Santana Aguiar e Ana Tereza Vasconcelos
EPI_ISL_471539	Hospital Universitario da USP Sao Paulo	Instituto Adolfo Lutz, Interdiciplinary Procedures Center, Strategic Laboratory	Claudio Tavares Sacchi, Claudia Regina Gonçalves, Erica Valessa Ramos Gomes
EPI_ISL_471541	Hospital Geral Santa Marcelina	Instituto Adolfo Lutz, Interdiciplinary Procedures Center, Strategic Laboratory	Claudio Tavares Sacchi, Claudia Regina Gonçalves, Erica Valessa Ramos Gomes
EPI_ISL_471545	Hospital Sao Paulo de Ensino da Unifesp	Instituto Adolfo Lutz, Interdiciplinary Procedures Center, Strategic Laboratory	Claudio Tavares Sacchi, Claudia Regina Gonçalves, Erica Valessa Ramos Gomes
EPI_ISL_471546	AMA DR Jose Soares Hungria	Instituto Adolfo Lutz, Interdiciplinary Procedures Center, Strategic Laboratory	Claudio Tavares Sacchi, Claudia Regina Gonçalves, Erica Valessa Ramos Gomes
EPI_ISL_471548	Hospital do Servidor Público Estadual Francisco Morato de Oliveira	Instituto Adolfo Lutz, Interdiciplinary Procedures Center, Strategic Laboratory	Claudio Tavares Sacchi, Claudia Regina Gonçalves, Erica Valessa Ramos Gomes
EPI_ISL_471549	Hospital Municipal Carmen Prudente	Instituto Adolfo Lutz, Interdiciplinary Procedures Center, Strategic Laboratory	Claudio Tavares Sacchi, Claudia Regina Gonçalves, Erica Valessa Ramos Gomes
EPI_ISL_471552	Hospital Sancta Maggiore	Instituto Adolfo Lutz, Interdiciplinary Procedures Center, Strategic Laboratory	Claudio Tavares Sacchi, Claudia Regina Gonçalves, Erica Valessa Ramos Gomes
EPI_ISL_471556	Pronto Socorro Jose Ibrahin	Instituto Adolfo Lutz, Interdiciplinary Procedures Center, Strategic Laboratory	Claudio Tavares Sacchi, Claudia Regina Gonçalves, Erica Valessa Ramos Gomes
EPI_ISL_471562, EPI_ISL_471581, EPI_ISL_471582	Hosp. Municipal Prof. Dr. Alípio Corrêa Netto	Instituto Adolfo Lutz, Interdiciplinary Procedures Center, Strategic Laboratory	Claudio Tavares Sacchi, Claudia Regina Gonçalves, Erica Valessa Ramos Gomes
EPI_ISL_471647	Hospital Municipal de Barueri Dr. Francisco Moran	Instituto Adolfo Lutz, Interdiciplinary Procedures Center, Strategic Laboratory	Claudio Tavares Sacchi, Claudia Regina Gonçalves, Erica Valessa Ramos Gomes
EPI_ISL_471648	UBS e Pronto Socorro Jd. Jacira	Instituto Adolfo Lutz, Interdiciplinary Procedures Center, Strategic Laboratory	Claudio Tavares Sacchi, Claudia Regina Gonçalves, Erica Valessa Ramos Gomes
EPI_ISL_473651	West of Scotland Specialist Virology Centre, NHSGGC / MRC-University of Glasgow Centre for Virus Research	COVID-19 Genomics UK (COG-UK) Consortium	Ana da Silva Filipe, Natasha Johnson, Kathy Smollett, Daniel Mair, Stephen Carmichael, Lily Tong, Jenna Nichols, Elihu Aranday-Cortes, Kirstyn Brunker, Yasmin Parr, Alice Broos, Kyriaki Nomikou; Sarah McDonald, Marc Niebel, Patawee Asamaphan; Richard Orton, Joseph Hughes, Sreenu Vattipally, David L Robertson; Alasdair MacLean, Rory Gunson; Kathy Li, Natasha Jesudason, Rajiv Shah, James Shepherd, Antonia Ho, Emma Thomson
EPI_ISL_476152, EPI_ISL_476156, EPI_ISL_476157, EPI_ISL_476159, EPI_ISL_476161, EPI_ISL_476162, EPI_ISL_476163, EPI_ISL_476165, EPI_ISL_476167, EPI_ISL_476169	Laboratório de Patologia Clínica - UNICAMP	Laboratório de Estudos de Vírus Emergentes - UNICAMP	José Luiz Proença-Modena, Magnun Nueldo Nunes dos Santos, Angelica Schreiber, Julia Forato,Camila Simeoni, Marcilio Jorge Fumagalli, Mariene Ribeiro Amorim, Darlan da Silva Candido, Nuno Rodrigues Faria, Julien Theze, Luiz Gonzaga,Jaqueline Goes Jesus e William Marciel de Souza
EPI_ISL_476244, EPI_ISL_476259	Hospital da Clínicas da Faculdade de Medicina da Universidade de São Paulo	Instituto de Medicina Tropical da Univesidade de São Paulo	Samples: Ingra Morales Claro, Erika Regina Manuli, Cecilia Salete Alencar, Carolina S. Lazar, Silvia F. Costa; Sequencing: Ingra Morales Claro, Jaqueline Goes de Jesus, Erika Regina Manuli, Flavia Cristina da Silva Sales, Thais de Moura Coletti, Camila Alves Maia da Silva, Mariana Severo Ramundo, Giula Magalhaes Ferreira, Darlan da Silva Candido, Julien Theze, Nuno Faria, Ester Sabino
EPI_ISL_476293, EPI_ISL_476312, EPI_ISL_476318, EPI_ISL_476321, EPI_ISL_476322	DB Diagnósticos do Brasil	Instituto de Medicina Tropical da Univesidade de São Paulo	Samples: Nelson Gaburo Jr; Sequencing: Ingra Morales Claro, Jaqueline Goes de Jesus, Erika Regina Manuli, Flavia Cristina da Silva Sales, Thais de Moura Coletti, Camila Alves Maia da Silva, Mariana Severo Ramundo, Giula Magalhaes Ferreira, Darlan da Silva Candido, Julien Theze, Nuno Faria, Ester Sabino
EPI_ISL_476337, EPI_ISL_476338,	Laboratório de Patologia Clínica - UNICAMP	Laboratório de Estudos de Vírus	José Luiz Proença-Modena, Magnun Nueldo Nunes dos Santos, Angelica Schreiber, Julia Forato,Camila Simeoni, Marcilio Jorge Fumagalli, Mariene Ribeiro Amorim, Darlan da Silva Candido, Nuno Rodrigues Faria, Julien Theze, Luiz Gonzaga,Jaqueline Goes

Section 3. Supplementary figures

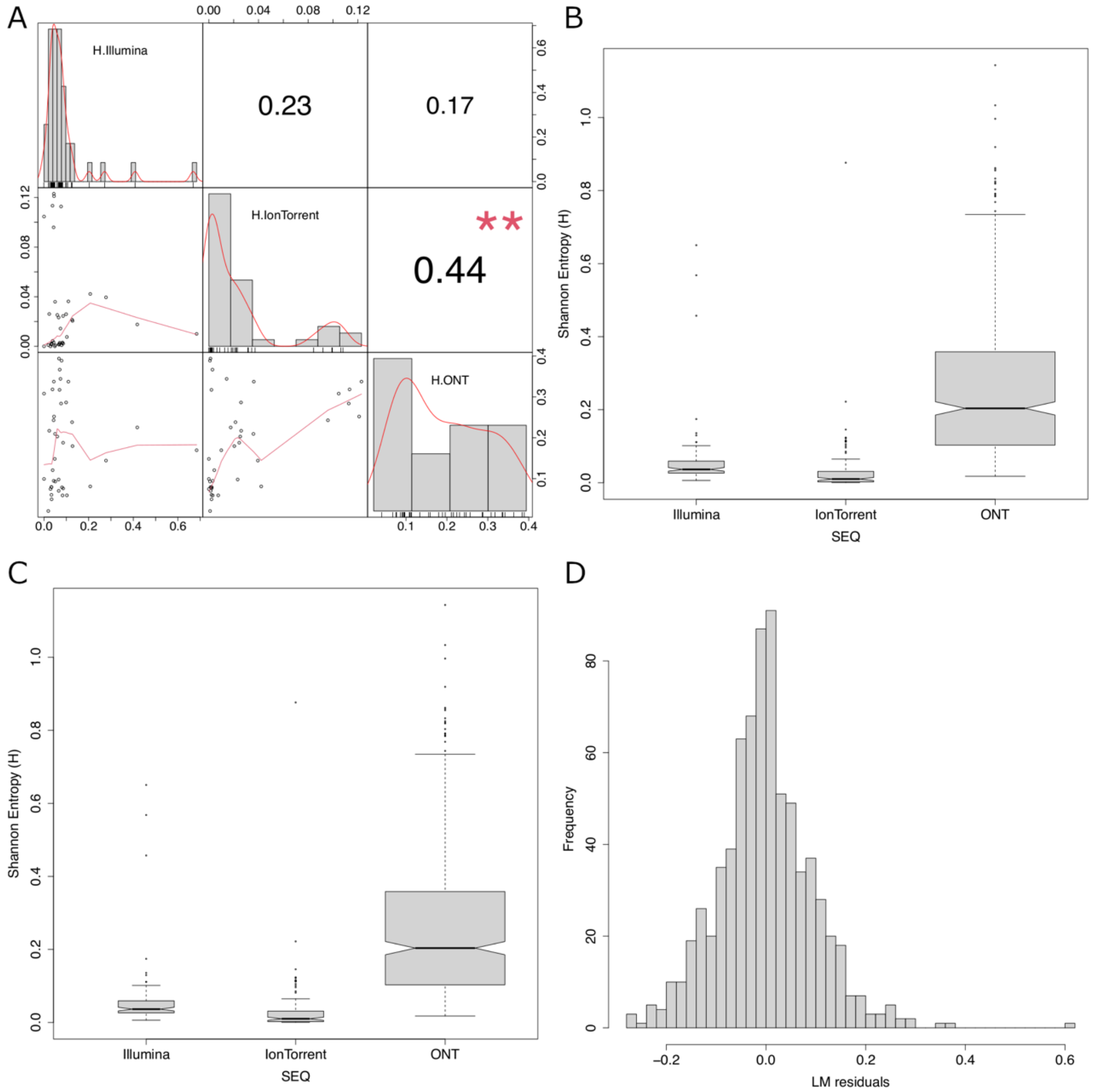

Figure S1. Effect of sequencing technology on intra-host diversity estimations. A) The samples were sequenced using three different technologies: Illumina, Ion Torrent, and ONT. In particular, samples RIV-M5, RIV-M11, RIV-M12, RIV-M13, RIV-M14, and RIV-M15 were sequenced with all three technologies. The graphic depicts a Spearman correlation matrix of the Shannon Entropy values (H) estimated from allele frequencies for each sequencing approach. Unexpectedly, the more error-prone ONT technology produces higher H values. This is clear when ONT is compared to IonTorrent data (rho=0.44, p-value <0.001), but less evident when including Illumina data in the comparisons. Entropy calculations for Illumina show a few high values, which could be due to differences in library design (which does not follow the Artic amplicon used elsewhere). B) For the entire sample set, after filtering observations with genome coverage < 100 reads, the data consisted of 148, 108 and 494 points obtained by Illumina,

Ion Torrent, and ONT sequencing, respectively. Boxplots show entropy distributions of H for each sequencing technology, H being higher for ONT. C) To better fit a normal distribution, H was square-root transformed (H exp 1⁄4). D) The histogram depicts the distribution of residuals from the linear model implemented (H exp 1⁄4 ~ SEQ + SAMPLE + MUTATION).

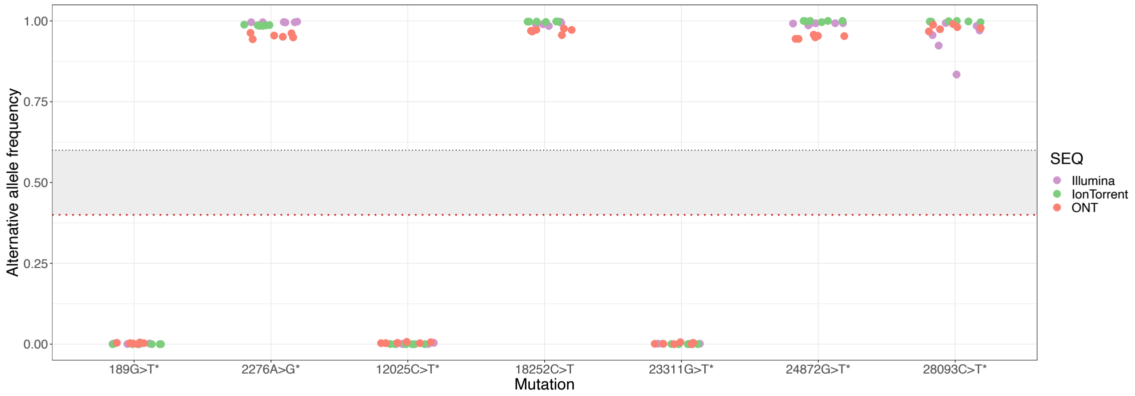

Figure S2: Effect of sequencing technology on within-host diversity analysis. For six samples (RIV-M5, RIV-M11, RIV-M12, RIV-M13, RIV-M14 and RIV-M15), SARS-CoV-2 genomes were obtained by Illumina, IonTorrent and ONT sequencing. Alternative allele frequencies for synapomorphies in the clade BR-UY-II33, as observed from trimmed bam files of these samples, are shown. Positions and annotation follow Wuhan's reference sequence MN908947.3. The asterisks indicate synapomorphic sites where the MUTATION explanatory variable shows a significant coefficient in the implemented linear model (p-value <= 0.05). In other words, the allele frequencies for these sites are robust to sequencing technology. Despite observing variability in the allele frequencies related to the sequencing strategy, where ONT values appear more dispersed, the absence of alleles with intermediate frequencies is notorious. In fact, synapomorphy 28093C>T may show a different behavior due to Illumina data points but still, allele frequencies do not reach intermediate frequencies. The gray zone in the plot shows the intermediate allele frequency range (40 - 60%) as defined by[20]. The authors also highlight that ONT sequencing enables detection of within-specimen single nucleotide variants from ~40% of allele frequency with adequate accuracy (dotted red line).

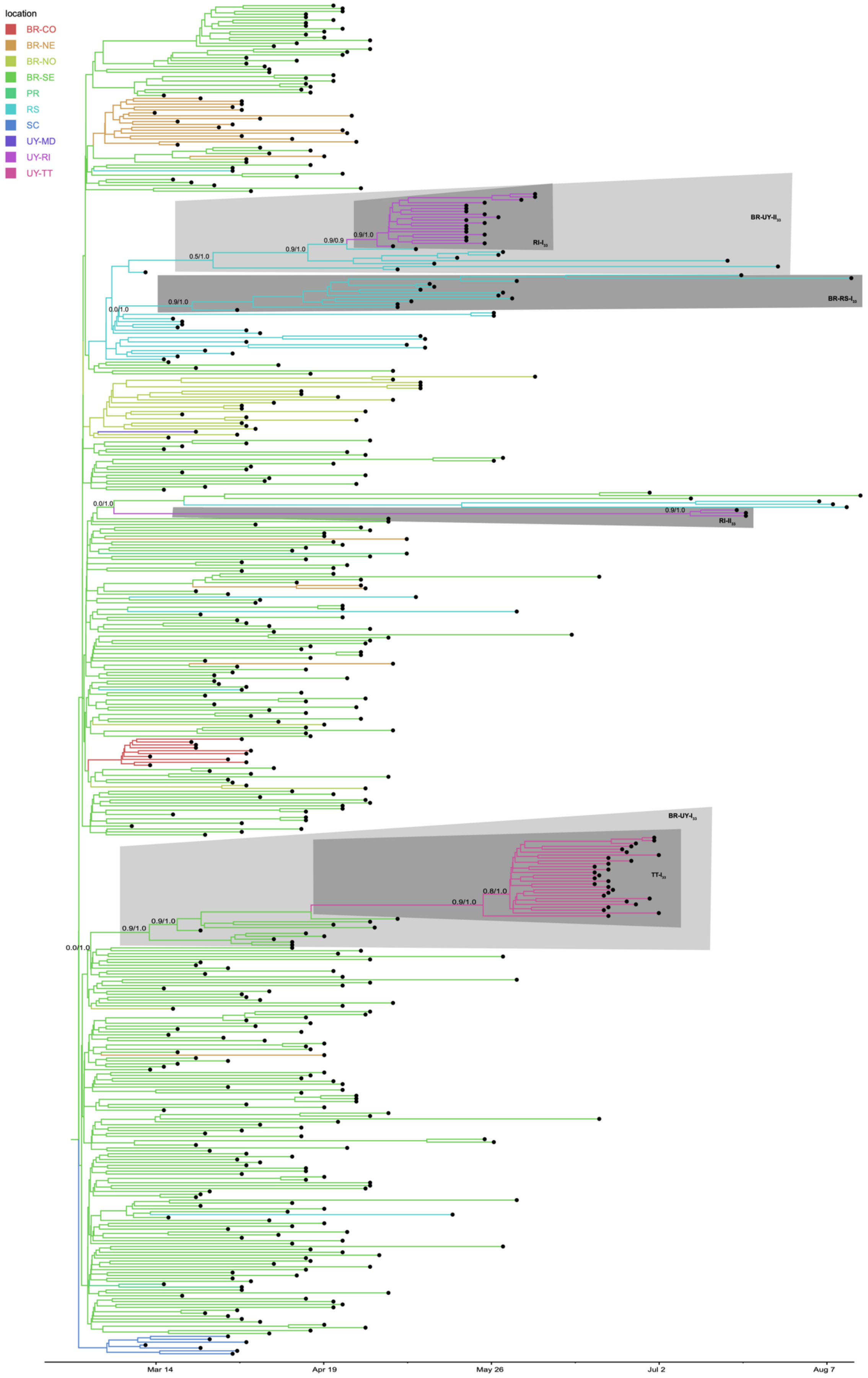
location
BR-CO
BR-NE
BR-NO
BR-SE
PR
RS
SC
UY-MD
UY-RI
UY-TT
BR-UY-II33
RI-I33
BR-RS-I33
RI-II33
BR-UY-I33
TT-I33
0.9/1.0
0.9/0.9
0.5/1.0
0.0/1.0
0.8/1.0
Mar 14
Apr 19
May 26
Jul 2
Aug 7

Figure S3. Spatiotemporal dissemination of SARS-CoV-2 B.1.1.33 Uruguayan-Brazilian variants. Time-scaled Bayesian phylogeographic MCC tree. Branches are colored according to the most probable location state of their descendant nodes as indicated at the legend. Posterior probability/Posterior state probability support values are indicated at key nodes.

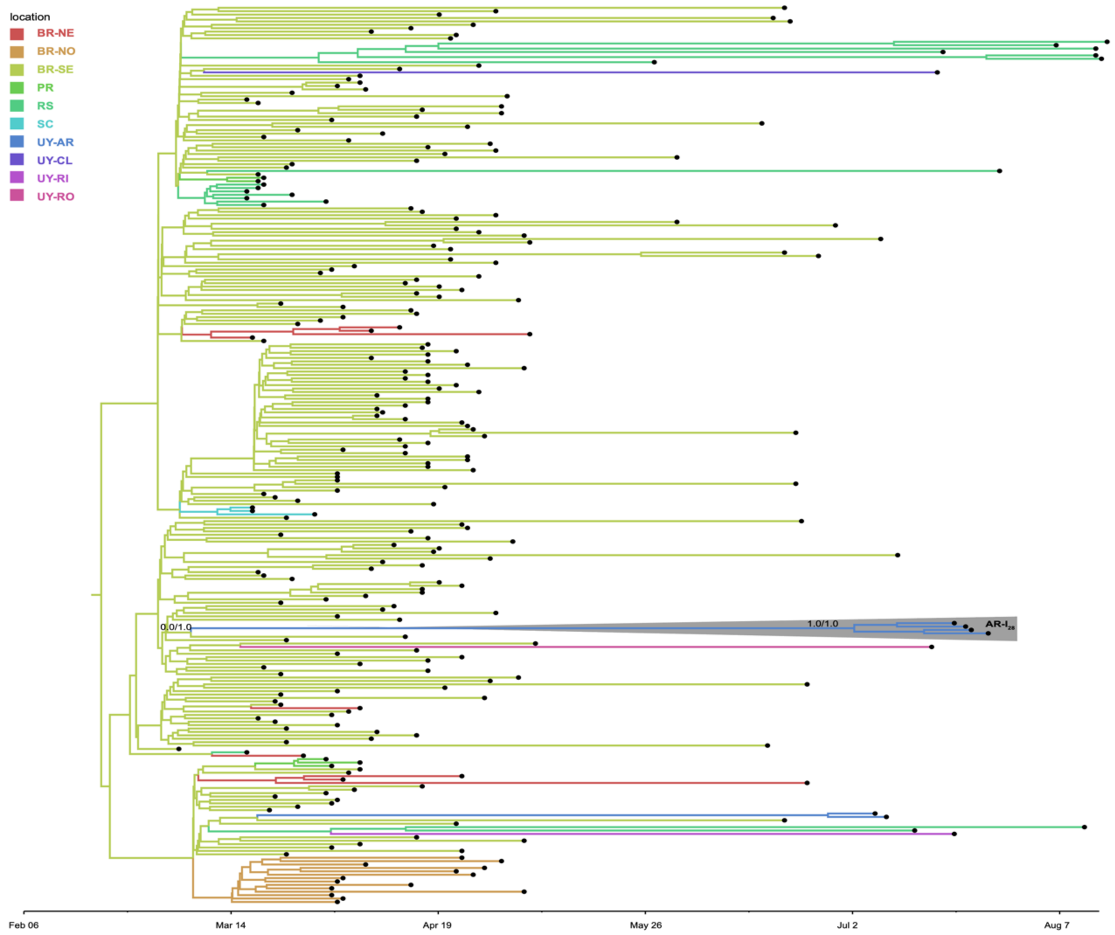

Figure S4. Spatiotemporal dissemination of SARS-CoV-2 B.1.1.28 Uruguayan-Brazilian variants. Time-scaled Bayesian phylogeographic MCC tree. Branches are colored according to the most probable location state of their descendant nodes as indicated at the legend. Posterior probability/Posterior state probability support values are indicated at key nodes.

Section 4. Other Supplementary Materials for this manuscript

4.1. Projects approval by the American Hospital ethics committee

Montevideo, 7 de julio de 2020

RESOLUCIÓN DEL COMITÉ DE ÉTICA EN LA INVESTIGACIÓN DE SANATORIO AMERICANO (CEI-SASA) SOBRE EL TRABAJO "VIGILANCIA EPIDEMIOLÓGICA DEL COVID-19 EN LAS FRONTERAS URUGUAYAS Y ANÁLISIS DE SU TRANSMISIÓN EN EL INTERIOR DEL PAÍS."

Introducción. Habiendo recibido este Comité de Ética de la Investigación de Sanatorio Americano (CEI-SASA) para su correspondiente análisis el trabajo: ***"Vigilancia epidemiológica del COVID-19 en las fronteras uruguayas y análisis de su transmisión en el interior del país.",*** siendo el patrocinante y, a la vez, centro investigador, el Instituto Pasteur de Montevideo (IPM), bajo la conducción de su Investigador Principal la Dra. Lucía Spangenberg, trabajando con la Investigadora Asistente y representante del Sanatorio Americano, la Dra. Verónica Noya, desea realizar las consideraciones insertas a continuación.

Características del Estudio. En primer lugar, se realiza una amplia y bien documentada justificación del estudio, fundamentándose en argumentos virológicas, y epidemiológicos.

En relación con los componentes virológicos de dicha justificación, se remarca que los virus de ARN se encuentran entre los agentes infecciosos de evolución más rápidos conocidos hasta el momento. Tal rasgo diferencial se halla condicionado por múltiples factores generadores de diversidad integrados a su ciclo replicativo, lo que determina altas tasas de mutación. En este tipo de patógenos de evolución rápida, la variabilidad en sus genomas a nivel poblacional ocurre en la misma escala de tiempo que los procesos epidemiológicos y ecológicos que determinan su éxito epidémico. Debido a esta superposición de escalas, diversos componentes de los procesos epidemiológicos y ecológicos que dan forma a los patrones genéticos pueden ser cuantificados mediante técnicas filogenéticas, acopladas a modelos poblacionales. Se puede así realizar una inferencia de cambios en el tamaño de la población, mapear el patrón de diseminación espacio-temporal del virus, caracterizar sus redes de transmisión, distinguir casos transmitidos localmente de importados, estimar el momento de su introducción a una determinada área geográfica o población y el posible origen geográfico de las diferentes variantes.

En referencia a los factores epidemiológicos, la actual pandemia de enfermedad por coronavirus (SARS-CoV-2) de 2019 (COVID-19), continúa en fase de rápida expansión que, aunque desigual según las regiones, ya afecta a millones de personas en todo el planeta, con cientos de miles de muertos. Existen muy diversas iniciativas de

investigación científica, tendientes a secuenciar los genomas de las cepas circulantes para así caracterizar cómo evoluciona y se propaga el agente. En consecuencia, es crucial la cooperación internacional amplia, con el mayor y más cristalino intercambio de información. De esta manera, se busca asegurar que el conocimiento generado se convierta en un bien público de uso global, erigida así como la mejor estrategia investigacional para el combate de la pandemia. ***Otro aspecto relevante es la posibilidad de analizar a partir de las secuencias virales, la existencia de variantes que generen cambios en la transmisibilidad viral (con implicancias en el manejo de la pandemia) y/o cambios en la virulencia y patogénesis viral (con implicancias en las intervenciones médicas e inmunológicas a los pacientes).***

A continuación, se describen las características geográficas y poblacionales de nuestras fronteras, remarcando en la "frontera seca" con el Brasil las denominadas "ciudades gemelas", así como las ciudades argentinas de las costas del Río Uruguay, unidas por puentes internacionales con ciudades de nuestro país. La gran intensidad y permeabilidad de nuestras relaciones trans-fronterizas vuelven una cuestión de extrema urgencia, la de reforzar la vigilancia sanitaria de las fronteras y realizar un monitoreo continuo de la circulación del virus en el interior de nuestro país.

Se relata la prontitud y alta eficacia colaborativa del sector académico, luego de decretada la emergencia sanitaria, con el rápido desarrollo de técnicas diagnósticas y el diseño de los equipos de protección personal y soporte ventilatorio, aprovechando la infraestructura y capacidades instaladas en los centros regionales de investigación de la UdelaR y del INIA. En este sentido, ***consideran los proponentes de este trbajo que el análisis de las secuencias genómicas virales provenientes de muestras del interior del país constituirá una herramienta poderosa para identificar en tiempo real los eventos de introducción y transmisión local del virus a partir de los países limítrofes,*** contribuyendo a la toma de decisiones en el manejo de la pandemia que realizan los actores gubernamentales, debidamente asesorados en el plano científico.

Los autores del proyecto se presentan con un grupo inter y trans-disciplinario a un llamado específico, con el nombre de *"Vigilancia epidemiológica del COVID-19 en las fronteras uruguayas y análisis de su transmisión local en el interior del país"*, con el cual titulan su protocolo de investigación. Dicha investigación será prospectiva y meramente observacional, desprovista de cualquier rasgo de invasividad, incluido el de tomar decisiones clínicas basadas en los resultados genómicos y dedicada a obtener las muestras para el análisis genómico correspondiente.

Objetivo General. El objetivo general del presente trabajo consiste en monitorear y caracterizar las variantes de SARS-CoV-2 que circulan en los departamentos del interior, con especial foco de interés en aquellos departamentos que tienen límite fronterizo con Argentina y Brasil.

Objetivos Específicos. Se describen varios objetivos específicos, relativos a la caracterización genómica de las diversas cepas identificadas, localizando su lugar de origen y sus vías de trasmisión, capacitando al personal involucrado, generando una red-país de laboratorios interconectados, así como contribuyendo a enriquecer el diálogo con las autoridades nacionales y colaborando así en la adecuada toma de decisiones.

Se proclama que *"el objetivo central de la descentralización del proceso de secuenciado es formar recursos humanos locales dentro de cada institución, tanto en el manejo de laboratorio, como en el uso de herramientas de análisis bioinformático posterior. Esta capacitación permitirá el desarrollo de investigaciones independientes implementando estas estrategias, tanto en el caso de futuras emergencias sanitarias como en futuros proyectos que se puedan ver beneficiados de usar esta nueva tecnología, fortaleciendo a su vez los vínculos entre distintos grupos de trabajo."*

Se detallan los distintos centros involucrados en el Proyecto con la integración de los diversos grupos de trabajo de cada centro.

Análisis filogenético. Para evaluar la epidemia de SARS-CoV-2 en Uruguay dentro de un contexto regional y global e inferir rutas de entrada a nuestro país, los análisis evolutivos incluirán, además de las secuencias aquí generadas, subconjuntos representativos de las secuencias disponibles en la base de datos GISAID EpiCoV de los países de la región y del mundo.

Consentimiento Informado. Los proponentes del estudio elaboran un Consentimiento Informado (CI) específico para el secuenciado del genoma viral y envío del proyecto para su aprobación por los respectivos comités de ética de cada centro de diagnóstico. En el mismo reproducen los elementos generales del estudio en un lenguaje que procura ser lo más accesible posible, dadas las complejidades específicas del mismo. Sin embargo, resulta claro que los investigadores, si pretenden ser completamente inteligibles para el futuro reclutado, deberán llevar a cabo una prolija y compleja tarea explicativa, bastante bien lograda desde el inicio, a nuestro entender, en el texto del propio CI. Se avisa de los cuidados brindados a las muestras y se anuncia su futura destrucción, una vez culminados los respectivos estudios genómicos. Se explicitan tanto la ausencia de beneficios directos para los reclutados como el posible aporte del estudio en el manejo de los brotes pandémicos. Se ratifica el carácter no invasivo del trabajo propuesto, incluyendo el descarte de la futura influencia de los datos obtenidos en las decisiones clínicas de la persona reclutada, como ya fue expuesto líneas arriba. Se ratifica la estricta confidencialidad de los datos identificatorios, basada en la ley de habeas data y se aclara que *"Solamente miembros del Comité Ético y autoridades regulatorias pueden tener acceso a esta información si así fuera*

necesario.", lo cual, aunque es de cierta vaguedad en cuanto a su alcance y condiciones de acceso en el manejo de los datos personales, podría resultar aceptable en el contexto del presente estudio.

Resolución. Luego de analizar las características del estudio denominado ***"Vigilancia epidemiológica del COVID-19 en las fronteras uruguayas y análisis de su transmisión en el interior del país.",*** debido a su trascendencia epidemiológica, que habrá de permitir una mejor planificación del enfrentamiento a la pandemia de Covid-19 en nuestro país, la urgencia en su pronta aprobación, dada la consiguiente morbilidad y la pérdida de vidas humanas, provocadas por la rápida expansión virósica, a lo que debe sumarse la formación de recursos humanos en la correspondiente red de laboratorios en condiciones de realizar análisis genómicos, la calidad del diseño metodológico y el prestigio tanto del centro proponente (IPM) como de sus investigadores, este CEI-SASA aprueba plenamente su realización en nuestra institución.

Por el Comité de Ética en la Investigación – Sanatorio Americano:

Dr Oscar Cluzet

Dr. Enrique Méndez

Dr. Alberto Aliaga

Sr. Richard Riera

References

[1] 10.2807/1560-7917.ES.2020.25.3.2000045

[2] dx.doi.org/10.17504/protocols.io.bfy7jpzn, BioRxiv

[3] https://dx.doi.org/10.17504/protocols.io.bdp7i5rn

[4] https://doi.org/10.1038/nprot.2017.066

[5] https://doi.org/10.1038/nprot.2017.066

[6] https://www.biorxiv.org/content/10.1101/2020.10.06.328328v1

[7] https://artic.network/ncov-2019/ncov2019-bioinformatics-sop.html, accessed on December 2020.

[8] https://www.nature.com/articles/nbt.3820

[9] https://github.com/iferres/ncov2019-artic-nf

[10] https://doi.org/10.1038/nmeth.1923

[11] https://doi.org/10.1093/nar/gkf436

[12] doi:10.1093/molbev/msx149

[13] doi: 10.1093/ve/vew007

[14] doi:10.1093/molbev/msi103

[15] doi: 10.1093/ve/veaa061

[16] https://virological.org/t/time-dependence-of-sars-cov-2-substitution-rates/542

[17] doi:10.1093/sysbio/syy032

[18] doi: 10.1002/j.1538-7305.1948.tb01338.x

[19] R Core Team (2020). R: A language and environment for statistical computing. R Foundation for Statistical Computing, Vienna, Austria. URL https://www.R-project.org/

[20] Bull, R.A., Adikari, T.N., Ferguson, J.M. *et al.* Analytical validity of nanopore sequencing for rapid SARS-CoV-2 genome analysis. *Nat Commun* **11,** 6272 (2020). https://doi.org/10.1038/s41467-020-20075-6
